# Evidence for a modulatory effect of IL-10 on both Th1 and Th2 cytokine production: The role of the environment

**DOI:** 10.1016/j.clim.2010.12.019

**Published:** 2011-04

**Authors:** Camila A. Figueiredo, Neuza M. Alcantara-Neves, Leila D. Amorim, Nivea B. Silva, Lain C. Pontes de Carvalho, Philip J. Cooper, Laura C. Rodrigues, Maurício L. Barreto

**Affiliations:** a Instituto de Ciências da Saúde, Universidade Federal da Bahia, Salvador, Bahia, Brazil; b Instituto de Matemática, Universidade Federal da Bahia, Salvador, Bahia, Brazil; c Centro de Pesquisas Gonçalo Moniz, Fundação Oswaldo Cruz—FIOCRUZ, Salvador, Bahia, Brazil; d Universidad San Francisco de Quito, Quito, Ecuador; e Centre for Infection, St George's, University of London, London, UK; f London School of Hygiene and Tropical Medicine, London, UK; g Instituto de Saúde Coletiva, Universidade Federal de Bahia, Salvador,Bahia, Brazil

**Keywords:** BMI, Body mass index, CpG-DNA, bacterial DNA, DC, dendritic cell, IFN, Interferon, LB, B lymphocyte, LPS, lipopolysaccharide, MΦ, macrophage, MHC, major histocompatibility complex, Neu, neutrophil, NO, nitric oxide, OR, odds ratio, PBLs, peripheral blood leukocytes, SCAALA, Social Changes Asthma and Allergy in Latin America, TGF-β, transforming growth factor β, Th1, T helper 1, Th17, T helper 17, Th2, T helper 2, TLR, toll like receptors, Treg, T regulatory, WBC, whole blood cells culture, IL-10, Th1/Th2, Environment, Sewage system, Street paving, Immune regulation

## Abstract

Allergic and other immune-mediated diseases are complex disease states determined by interplay between host genetics and environmental factors. Environmental changes such as fewer infections and reduced exposure to microbial products have been suggested to have led to insufficient regulation of Th1 and Th2 immune responses, causing an increased incidence of inflammatory diseases. The objective of the present study was to investigate the effect of poor living environmental conditions on mitogen-induced production of cytokines (Th1 and Th2) by peripheral blood leukocytes in children living in urban Brazil and investigate the role of IL-10 in modifying this effect. Our data showed that the proportion of children producing Th1 and Th2 cytokines was lower among those with poor living conditions and that this finding was stronger in children producing IL-10. These results provide a possible biologic explanation for the temporal trends of increasing risk of inflammatory diseases observed in populations living in affluent countries.

## 1 Introduction

Allergic and other immune-mediated diseases are complex disease states that are considered to be determined by interactions between host genetic and environmental risk factors [1–3]. The rapid change in the prevalence of these diseases observed in affluent countries over the last 40 years or so has been attributed largely to environmental changes [4,5].

The “hygiene hypothesis”, originally proposed by Strachan [6], has explained the temporal trends in the prevalence of allergic disease in terms of decreased exposures to microbes in the environment during childhood as a consequence of modern health care interventions (e.g. antibiotics and vaccines) and improved environmental hygiene within and outside the home [7]. As a consequence, the protection against allergic diseases provided by microbial exposures may not be sufficient in populations living in affluent countries and may have led to a failure to develop robust immune regulatory mechanisms [8,9] that are necessary for controlling the inflammation that predisposes individuals to the development of allergy and other inflammatory diseases such as diabetes, also on the rise in many affluent countries [10]. We have previously demonstrated that children living in circumstances of poor hygiene, without access to sanitation or clean water during the first three years of life, have elevated spontaneous production of IL-10 later in life, and we have speculated that microbial exposures could be responsible for this modulation [11].

In the present study, we investigated the effects of environmental exposures, many related to poor sanitary conditions, on the production of cytokines by mitogen-stimulated peripheral blood leukocytes (PBLs) in children aged 4–11 years living in poverty in the city of Salvador, Northeastern Brazil. To our knowledge, our data provides the first evidence that poor living conditions are inversely associated with both Th1 and Th2 cytokine production and that this effect might be modified by IL-10.

## 2 Objective

The objective of the present study was to investigate the effect of poor living environmental conditions on mitogen-induced production of cytokines (Th1 and Th2) by peripheral blood leukocytes in children living in urban Brazil and investigate the role of IL-10 in modulating this effect.

## 3 Materials and methods

### 3.1 Study population and data collection

This study was conducted in the city of Salvador in Northeastern Brazil, which has a population of 2.8 million and has a high prevalence of wheezing (31.22%), atopy (37.96%) and parasite infections (*A. lumbricoides* and *T. trichiura*, 16.99% and 11.58%, respectively). The design of this study has been reported elsewhere [5,12]. In short, the study population included 1445 children who were recruited in infancy for a prospective study measuring the impact of a citywide sanitation program on childhood morbidity [13]. We collected data from children born between 1994 and 2001 who lived in sentinel neighborhoods in the city. We administered standardized questionnaires to the children's guardians between 1997 and 2003 (baseline) and collected data on demographic and social variables as well as observations on the home environment by the same time [14]. Data on access to sanitation was collected in 2000 — the presence of a sewage system defined by the connection of the household to the sewage system. Also in 2000, the presence of street paving where the child's household was located was used as a marker of exposure to asphalt *vs* dirt roads. Data collection was repeated in 2005, at which time blood samples were collected. Of the 1445 children included in the study, we obtained cytokine data in 2005 from 1006 children for IFN-γ, 1356 for IL-10, 1289 for IL-13, and 1243 for IL-5. The present analysis included all the 793 children for whom complete data (cytokines levels, environmental and socioeconomics characteristics) were available.

### 3.2 Blood collection and whole blood culture

We collected venous blood into heparinized tubes and cultured the blood at a dilution of 1:4 in RPMI (Gibco, Auckland, New Zealand) containing 10 mM glutamine (Sigma-Aldrich, St. Louis, MO, USA) and 100 μg/mL gentamicin (Sigma-Aldrich, St. Louis, MO, USA). The cell cultures were started within 6 h of blood collection and were maintained in a humidified environment of 5% CO_2_ at 37 °C for 24 h for detection of IL-10 and for 5 days for the detection of IL-13, IL-5, and IFN-γ in the presence of pokeweed mitogen (PWM; Sigma-Aldrich, St. Louis, MO, USA) (2.5 μg/mL) or media alone (spontaneous production).

### 3.3 Cytokine production

We measured the production of Th2 (IL-5 and IL-13), Th1 (IFN-γ), and Treg (IL-10) cytokines in whole blood culture supernatants using commercially available antibody pairs and recombinant cytokine standards (BD Pharmingen, San Diego, CA, USA) by sandwich ELISA, according to the manufacturer's instructions. Cytokine concentrations were determined by interpolation of standard curves. The detection limits (low/high) for each cytokine were: 15.6/500 pg/mL, 62.5/4000 pg/mL, 18.5/300 pg/mL, and 31.3/500 pg/mL for IL-5, IL-13, IFN-γ, and IL-10, respectively. Responders were defined as those children with cytokine concentrations above the lower detection limits after subtracting negative control values (basal cytokine production).

### 3.4 Statistical analyses

Descriptive statistics were presented for all variables considered in this study. Geometric means and corresponding 95% confidence intervals for IFN-γ, IL-5 and IL-13 are presented in graphs. The associations between cytokine production and environmental characteristics were evaluated by calculation of odds ratios (OR) using logistic regression models. The odds ratios were used to compare the chance of producing cytokines among those with worse environmental characteristics to the chance of producing cytokines among those with improved situation. ORs were adjusted for child's sex, age and nutritional status, and maternal schooling. Analyses stratified by IL-10 responsiveness were done using logistic regression models. Adjusted ORs and 95% confidence intervals were presented. Interaction effects between IL-10 and environmental characteristics on Th1 (IFN-γ) and Th2 (IL-5 and IL-13) cytokine production from PWM-stimulated cultures were tested using Wald test with significance level at 0.05. Statistical analyses were performed using STATA v.10 software.

### 3.5 Ethical considerations

We obtained ethical approval for this study from the Brazilian National Ethical Committee in 2004. Written informed consent was obtained from the legal guardian of each child.

## 4 Results

### 4.1 Description of study population

Table 1 contains the demographic characteristics of the study population as well as the environmental variables measured. The majority of the study population was constituted of boys (52.6%), was aged 6–7 years (40.4%) and classified as eutrophic according to BMI (87.9%). Almost half (44.5%) of the mothers had completed high school or college education. 86% of the population had access to tap water, and 76.3% had home garbage collection daily or at least three times a week; 65.1% had paved streets and 83.6% had adequate bathroom conditions. With respect to a household sewage system, we obtained data from two different time points: early life (i.e. < 3 years old) and later childhood (age range, 4–11 years). Of the children surveyed, 57.4% never had sewage system or at most had it in only one of the time periods (early or late childhood), and 42.6% had a sewage system at both time points.

### 4.2 The environment effect on Th1 and Th2 cytokine production

**Figure 1:**
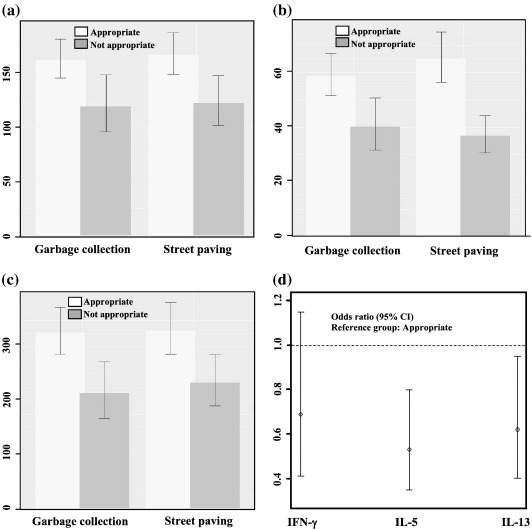
Cytokine suppression as represented by geometric means for IFN-γ (a), IL-5 (b) and IL-13 (c) concentrations (pg/mL) according to garbage collection and street paving and (d) Odds ratio and 95% confidence interval for cytokine responsiveness and combined living conditions comparing to reference group (children who had appropriated living conditions).

Table 2 presents the estimated effects of environmental exposures on Th1 and Th2 cytokine production. Poor living conditions were associated with reduced levels of Th1 and Th2 cytokines. Children who lived in houses with less frequent garbage collection and those whose households are allocated in areas without street paving were significantly less likely to have detectable levels of IFN-γ, IL-5 and IL-13 after adjustment for child's gender, age and nutritional status, and maternal schooling (a proxy for hygiene awareness). Similarly, poor sewage access was significantly associated with less frequent IL-5 production. There were non-significant associations between the other exposures (tap water, sewage system and bathroom condition), and the production of Th1 and Th2 cytokines (Table 2). The same effect of environmental factors was observed using cytokine's concentrations as presented in Figures 1(a–c). The geometric means for IFN-γ, IL-5 and IL-13 concentrations were much lower when garbage collection was not frequent and when street paving is not present where the children live (Figs. 1a–c). In addition, in order to estimate a combined effect of environmental exposures we compared (i) a group that refers the improved situation for all living conditions; and (ii) a group that refers having a poorer situation for at least one of those living conditions. We estimated the odds ratios and corresponding 95% confidence intervals for IFN-γ, IL-5 and IL-13 responsiveness for those groups. The results pointed out for a reduction of the production of all cytokines for the poorer group compared to the group with appropriate living conditions (Fig. 1d). For instance, the odds of producing IL-5 in the group with poorer situation for at least one of the living conditions is 47% smaller than that observed for the group with appropriate situation for all living conditions (OR = 0.53; 95%CI = 0.35,0.80).

### 4.3 Effect of IL-10 on relationship between environment and Th1 and Th2 cytokine production

Table 3 presents the results for a stratified analysis according to IL-10 responsiveness (non-responders *vs* responders). The relationship between the measured environmental exposures and the production of both Th1 and Th2 cytokines was affected by IL-10 responsiveness. It has been observed that, in general, the chance of producing Th1 and Th2 cytokines is strongly reduced among IL-10 responders, even though not always statistically significant. This reduction was observed in both Th1 and Th2 cytokine levels for the variables garbage collection, lack of sewage system in household and houses in street without paving. For instance, children who lived in households without connection to the sewage system at all or had it in only one of the time periods (early or late childhood), compared to those who always had sewage system, had significantly reduced production of IFN-γ (OR = 0.40; 95% OR = 0.2–0.8), IL-5 (OR = 0.44; 95% OR = 0.3–0.6) and IL-13 (OR = 0.47; 95% OR = 0.3–0.7) among IL-10 responders, On the other hand, among the IL-10 non-responders, there was a marked and significant increase in the association between presence of sewage system and the production of all these three cytokines (Table 3). The interaction effects between IL-10 response and sewage system were highly significant (*p*-values = 0.002, 0.002 and 0.001, respectively, for IFN-γ, IL-5 and IL-13). Likewise, children who were IL-10 responders and lived in households with deficient garbage collection or situated in unpaved streets showed significant reductions in the production of IFN-γ, IL-5 and IL-13. In addition, significant interaction effects between IL-10 response and street paving were also found for the production of Th1 and Th2 cytokines (*p*-values = 0.02, 0.003 and 0.003, respectively, for IFN-γ, IL-5 and IL-13).

## 5 Discussion

A biologic explanation for the temporal trends of increased prevalence of Th1 and Th2 inflammatory diseases has been provided by a reworking of the hygiene hypothesis reducing the emphasis of a Th2 *vs* Th1 immune bias, to emphasize the importance of a reduction of immune regulatory mechanisms to control Th1 and Th2 inflammatory responses [8,9]. The induction of such regulatory mechanisms, exemplified by elevated production of the immune regulatory cytokine IL-10, has been attributed to intense microbial exposures occurring during childhood — the decline in such exposures in many populations living in affluent countries and the increase in the prevalence of inflammatory diseases is considered to be a consequence of a failure in microbe-induced immune regulation [8,9]. However, to date little data to support such a hypothesis has been available from human populations. The data from the present study provides, to our knowledge, the first evidence from a population-based study that environmental factors associated with hygiene (i.e. garbage disposal, sewage, and street paving) are inversely associated with the production of both Th1 and Th2 cytokines. Such effects were greater among children producing IL-10 compared to those not producing IL-10, indicating that IL-10 may modulate these effects.

Over the past 20 years, a significant amount of *in vitro* and *in vivo* data have accumulated from experimental animal models and humans showing that Th1 and Th2 responses are reciprocally regulated [15], a process known as immune deviation. Thus, IL-12, IL-18, IFN-γ and IFN-α not only favor the development of Th1 cells but also inhibit the development of Th2 cells. On the other hand, Th2 type cytokines were believed to suppress Th1 cells [16,17]. Interleukin-10 (IL-10) was first described as cytokine synthesis inhibitory factor (CSIF), which was produced by mouse Th2 cells and inhibited activation and cytokine production by Th1 cells [18]. The ability of IL-10 to inhibit cytokine production by both T cells and NK cells was found to be indirect, via inhibition of accessory cell (macrophage/monocyte) function [19]. These studies were soon extended to show that IL-10 profoundly inhibited a broad spectrum of activated macrophage/monocyte functions, including monokine synthesis, NO production, and expression of class II MHC and costimulatory molecules such CD80/CD86 [19]. *In vitro* and *in vivo* studies with recombinant cytokine and neutralizing antibodies revealed pleiotropic activities of IL-10 on B, T, and mast cells [20–24], and provided evidence for *in vivo* significance of several of the IL-10 *in vitro* activities [25–27].

The significance of these experimental observations have emerged with the possible therapeutically use of IL-10 in humans to treat inflammatory disease states [28] and a mechanism to explain the increased prevalence of inflammatory diseases [6]. Alterations in regulatory cytokine levels, such as IL-10 and TGF-β, are believed to play an important role in mediating this last phenomenon [9]. We have previously demonstrated that children living in circumstances of poor hygiene without access to sanitation or clean water during the first three years of life have elevated spontaneous production of IL-10 later in life [11]. In the present work, we demonstrated that the effect of exposures to “dirt”, leading to reduction in the production of Th1 and Th2 cytokines by mitogen-stimulated PBLs, is related to IL-10. Among IL-10 responders, the ORs for the production of IFN-γ, IL-13 and IL-5 by PBLs were significantly smaller than those of non-responders for several of the variables related to increased “dirt” exposure investigated in this work. Further, significant interaction effects between IL-10 and sewage system or street paving were observed on Th1 and Th2 cytokine production. The data indicate that children living in houses in an environment of poor hygiene and low socioeconomic status were less likely to produce Th1 and Th2 cytokines and this effect is more marked in those with up-regulated IL-10.

The mechanisms by which environmental factors up-regulate IL-10 are unknown but, as we and others have previously hypothesized, exposures to pathogens, including parasites, might be relevant [11,29,30]. Work by our group has shown that a city-wide sanitation program, developed in the same setting that the present work was conducted, reduced significantly the occurrence of diarrhea [13] and the prevalence and incidence of intestinal helminthes [31]. Such findings can be related with our results, whereby the absence of sewage system seems to have the greater effect on cytokine modulation, a fact that may be explained by greater exposure to infections. We and others have previously demonstrated that parasites such as *Ascaris lumbricoides* and *Trichuris trichiura* can modulate immune response inducing hyporesponsiveness of PBLs to mitogen stimulation by activation of a regulatory network [29,32]. In this way, children who live in poor living conditions are more likely to become infected with potent immune modulators such as helminthes, viruses, bacteria and their products such as LPS, superantigens, peptidoglycans, bacterial CpG-DNA motifs and heat shock proteins, which may induce activation of a regulatory network. There are also two other possibilities related to this immune attenuation observed: (a) the attenuation is consequent to a primary immune activation event, i.e., persistence immune activation could lead to “immune exhaustion” with reduced cellular responsiveness to certain stimuli, as suggested in the antigen-rich intestinal mucosal compartment, mainly induced by aggressive pathogens; and (b) a pathogen-mediated inflammatory response induces recruitment of pro-inflammatory cells to the tissue at the site of the pathogenic challenge and thus dilutes the presence and detection of these elements in the peripheral blood. In addition, the immune modulation may be directly related to other poverty-associated factors, such as stressful life style and poor sleep conditions, which would coexist, in the same poor subpopulation, with the bad hygiene-associated factors that were studied in the present work. However, one likely hypothesis to explain our findings is that the lack of hygiene can lead a greater exposition to weakly pathogenic organisms, which can activate a regulatory network characterized by the up-regulation of Treg cells and increased production of IL-10 [33,34], which can be associated to infections with low parasite loads such as with *A. lumbricoides* and *T. trichuris*, which have been previously described infecting the studied population, as mentioned above [32].

**Figure 2:**
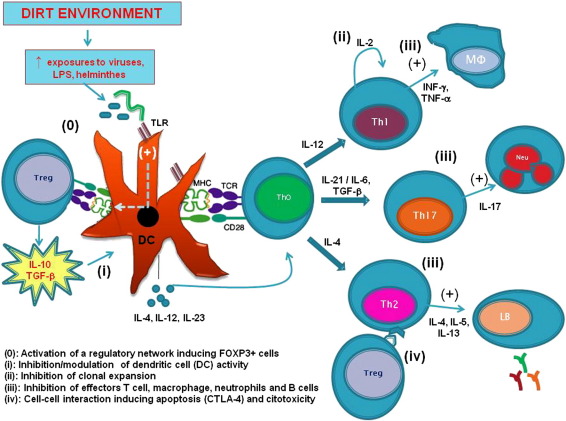
Regulatory network activated by environmental factors. Lack of hygiene and low socioeconomic status are likely to be associated with greater exposures to microbial products (e.g. LPS), and pathogens including helminth parasites which act through dendritic cells (DC) to activate CD4^+^CD25^+^FOXP3^+^ T cells (Treg) (0). The activation of Treg may suppress immune responses through 4 mechanisms: (i) Inhibition/modulation of DC activity through IL-10 and TGF-β; (ii) Inhibition of clonal expansion through competition for T cell growth factors such as IL-2; (iii) Inhibition of effectors cells, such as T cell, macrophage, neutrophils and B cells through IL-10 and TGF-β; and (iv) cell–cell interaction inducing apoptosis (CLTA-4) or cytotoxicity mediated by perforins and granzymes.

The activation of T regulatory cells (Fig. 2, (0)) might down-modulate the immune response through the following mechanisms: (i) Inhibition/modulation of dendritic cell activity through IL-10 and TGF-β; (ii) Inhibition of clonal expansion of T cells through competition for T-cell growth factors such as IL-2; (iii) Inhibition of effectors cells, such as T cell, macrophage, neutrophils and B cells through IL-10 and TGF-β; and (iv) cell–cell interaction inducing apoptosis (through CTLA-4) or cytotoxicity mediated by perforins and granzymes [35,36]. All these four mechanisms would be expected to be associated with reduced Th1 and Th2 cytokine production. However, the data presented herein, which show an association of exposure to “dirt” and high IL-10 production with a reduction of Th1 and Th2 cytokine production, would lend support to the first and third mechanisms, i.e., the exposure to “dirt” would ultimately lead to IL-10 production, which in its turn would directly suppress dendritic and type 1 and type 2 cytokine-producing T cells.

In summary, the present study provides evidence that poor living conditions are associated with reduced levels of both Th1 and Th2 cytokine production by PBLs, that this effect was associated to IL-10, and that it may explain why immune-mediated diseases may be less frequent in non-affluent countries.

## Figures and Tables

**Table 1:** Characteristics of the study population.

Variables	*n* (%)
Sex	
Female	376 (47.4)
Male	417 (52.6)
Age (years)	
4–5	207 (26.1)
6–7	320 (40.4)
8–11	266 (33.5)
Maternal education	
High school or college	353 (44.5)
Middle school	268 (33.8)
Elementary school	172 (21.7)
Tap water	
Yes	682 (86.0)
No	111 (14.0)
Garbage collection	
Daily or ≥ 3 days/week	608 (76.3)
< 3 days/week	188 (23.7)
Sewage system	
Always	338 (42.6)
Never or at most in one time point	455 (57.4)
Street paving	
Paved	516 (65.1)
Unpaved	277 (34.9)
Bathroom condition	
Adequate	663 (83.6)
Inadequate	130 (16.4)
Nutritional status	
Eutrophic	696 (87.8)
Overweight	97 (12.2)

**Table 2:** Effects of environmental and socioeconomics characteristics on Th1 (IFN-γ) and Th2 (IL-5 and IL-13) cytokine production by mitogen-stimulated peripheral blood leukocytes adjusted for child's sex, age, nutritional status, maternal schooling and for each environmental variables⁎.

Variables	*n*	IFN-γ		IL-5		IL-13	
		OR_crude_ (95% CI)	OR_adjusted_ (95% CI)	OR_crude_ (95% CI)	OR_adjusted_ (95% CI)	OR_crude_ (95% CI)	OR_adjusted_ (95% CI)
Overall (*n* = 793)							
Tap water							
Yes (*reference group*)	682	1.00	1.00	1.00	1.00	1.00	1.00
No	111	0.78 (0.46–1.34)	0.98 (0.49–1.96)	0.75 (0.49–1.16)	1.06 (0.61–1.84)	0.78 (0.49–1.24)	1.19 (0.66–2.14)
Garbage collection							
Daily or ≥ 3 days/week (*reference group*)	605	1.00	1.00	1.00	1.00	1.00	1.00
< 3 days/week	188	**0.49** **(0.32–0.75)**	**0.59** **(0.38–0.92)**	**0.49** **(0.35–0.70)**	**0.58** **(0.40–0.83)**	**0.53** **(0.37–0.76)**	**0.62** **(0.42–0.91)**
Sewage system							
Always (*reference group*)	338	1.00	1.00	1.00	1.00	1.00	1.00
Never or at most in one point	455	0.82 (0.55–1.24)	1.09 (0.70–1.70)	**0.61** **(0.44–0.85)**	0.73 (0.51–1.04)	**0.69** **(0.48–0.97)**	0.82 (0.57–1.20)
Street paving							
Paved (*reference group*)	516	1.00	1.00	1.00	1.00	1.00	1.00
Unpaved	277	**0.50** **(0.33–0.75)**	**0.57** **(0.37–0.88)**	**0.53** **(0.39–0.73)**	**0.66** **(0.47–0.93)**	**0.61** **(0.43–0.85)**	**0.74** **(0.51–1.07)**
Bathroom condition							
Adequate (*reference group*)	663	1.00	1.00	1.00	1.00	1.00	1.00
Inadequate	130	0.74 (0.45–1.22)	0.85 (0.45–1.63)	0.69 (0.46–1.03)	0.83 (0.49–1.39)	0.60 (0.42–0.98)	0.71 (0.41–1.21)

⁎ Bold means statistically significant results.

**Table 3:** Adjusted analysis for effects of environmental and socioeconomics characteristics on Th1 (IFN-γ) and Th2 (IL-5 and IL-13) cytokine production from mitogen-stimulated peripheral blood leukocytes stratified by IL-10 responsiveness⁎.

Variables	*n*	IFN-γ		IL-5		IL-13	
		IL-10 non-responders (*n* = 72)	IL-10 responders (*n* = 721)	IL-10 non-responders (*n* = 72)	IL-10 responders (*n* = 721)	IL-10 non-responders (*n* = 72)	IL-10 responders (*n* = 721)
		OR (95% CI)	OR (95% CI)	OR (95% CI)	OR (95% CI)	OR (95% CI)	OR (95% CI)
Overall (*n* = 793)							
Tap water							
Yes (*reference group*)	682	1.00	1.00	1.00	1.00	1.00	1.00
No	111	0.95 (0.16–5.59)	0.71 (0.33–1.53)	2.01 (0.29–13.99)	0.78 (0.48–1.29)	0.82 (0.14–4.69)	0.87 (0.50–1.51)
Garbage collection							
Daily or ≥ 3 days/week (*reference group*)	605	1.00	1.00	1.00	1.00	1.00	1.00
< 3 days/week	188	0.85 (0.18–3.93)	**0.29** **(0.16–0.51)**	0.39 (0.06–2.67)	**0.44** **(0.30–0.65)**	0.56 (0.12–2.57)	**0.47** **(0.31–0.72)**
Sewage system							
Always (*reference group*)	338	1.00	1.00	1.00	1.00	1.00	1.00
Never or at most in one time point	455	**12.48** **(1.5–106.7)**	**0.40** **(0.2–0.8)**	**7.44** **(1.1–49.0)**	**0.44** **(0.3–0.6)**	**5.48** **(1.3–23.8)**	**0.47** **(0.3–0.7)**
Street paving							
Paved (*reference group*)	516	1.00	1.00	1.00	1.00	1.00	1.00
Unpaved	277	1.41 (0.38–5.16)	**0.28** **(0.16–0.50)**	4.21 (0.91–19.42)	**0.43** **(0.30–0.63)**	3.03 (0.88–10.38)	**0.49** **(0.33–0.73)**
Bathroom condition							
Adequate (*reference group*)	663	1.00	1.00	1.00	1.00	1.00	1.00
Inadequate	130	0.70 (0.12–4.06)	0.89 (0.41–1.92)	1.55 (0.22–10.68)	0.78 (0.49–1.26)	0.58 (0.10–3.27)	0.76 (0.45–1.27)

⁎ Bold means statistically significant results.
